# Analyses of Lipid A Diversity in Gram-Negative Intestinal Bacteria Using Liquid Chromatography–Quadrupole Time-of-Flight Mass Spectrometry

**DOI:** 10.3390/metabo11040197

**Published:** 2021-03-26

**Authors:** Nobuyuki Okahashi, Masahiro Ueda, Fumio Matsuda, Makoto Arita

**Affiliations:** 1 Department of Bioinformatic Engineering, Graduate School of Information Science and Technology, Osaka University, 1-5 Yamadaoka, Suita, Osaka 565-0871, Japan; fmatsuda@ist.osaka-u.ac.jp; 2 Osaka University Shimadzu Analytical Innovation Research Laboratory, Graduate School of Engineering, Osaka University, 2-1 Yamadaoka, Suita, Osaka 565-0871, Japan; 3 Laboratory for Metabolomics, RIKEN Center for Integrative Medical Sciences, Yokohama, Kanagawa 230-0045, Japan; masahiro.ueda@riken.jp; 4 JSR-Keio University Medical and Chemical Innovation Center, JSR-Keio University, Shinjuku-ku, Tokyo 160-0016, Japan; 5 Division of Physiological Chemistry and Metabolism, Graduate School of Pharmaceutical Sciences, Keio University, Minato-ku, Tokyo 105-8512, Japan; 6 Cellular and Molecular Epigenetics Laboratory, Graduate School of Medical Life Science, Yokohama City University, Yokohama, Kanagawa 230-0045, Japan

**Keywords:** lipid A, LC-QTOF/MS, intestinal bacteria, *Escherichia coli*, Bacteroidetes

## Abstract

Lipid A is a characteristic molecule of Gram-negative bacteria that elicits an immune response in mammalian cells. The presence of structurally diverse lipid A types in the human gut bacteria has been suggested before, and this appears associated with the immune response. However, lipid A structures and their quantitative heterogeneity have not been well characterized. In this study, a method of analysis for lipid A using liquid chromatography–quadrupole time-of-flight mass spectrometry (LC-QTOF/MS) was developed and applied to the analyses of *Escherichia coli* and Bacteroidetes strains. In general, phosphate compounds adsorb on stainless-steel piping and cause peak tailing, but the use of an ammonia-containing alkaline solvent produced sharp lipid A peaks with high sensitivity. The method was applied to *E. coli* strains, and revealed the accumulation of lipid A with abnormal acyl side chains in knockout strains as well as known diphosphoryl hexa-acylated lipid A in a wild-type strain. The analysis of nine representative strains of Bacteroidetes showed the presence of monophosphoryl penta-acylated lipid A characterized by a highly heterogeneous main acyl chain length. Comparison of the structures and amounts of lipid A among the strains suggested a relationship between lipid A profiles and the phylogenetic classification of the strains.

## 1. Introduction

Lipopolysaccharide (LPS), a characteristic structure of Gram-negative bacteria, consists of a polysaccharide, core sugar, and lipid A moiety. Of these, lipid A is an active molecule that triggers inflammation via Toll-like receptor 4 (TLR4) in mammalian cells. The best characterized lipid A of *E. coli* has six acyl chains and two phosphate groups and is a potent agonist of TLR4 [1]. The structure of lipid A has been studied in various gram-negative bacteria [1,2]. Interestingly, the reported differences in the structure of lipid A (namely, the length of the acyl chain, number of acyl groups, number of phosphate groups, and their modifications) have been reported to be involved in the agonistic and antagonistic activity of TLR4 [2,3].

There are many types of Gram-negative commensal bacteria in the human intestine, most of which belong to two phyla, Bacteroidetes and Proteobacteria. Disturbance of microbial composition including these bacteria is called dysbiosis. It has been suggested that unusual microbiota induce TLR4 signaling, leading to certain diseases, inflammatory bowel disease (IBD), colitis-associated cancer, diabetes, Alzheimer’s disease, and Parkinson’s disease [4,5,6,7,8,9]. For example, the fecal microbiome of IBD patients has shown reduced diversity of Bacteroidetes and a significant increase in *E. coli* [5]. Interestingly, recent studies demonstrated that crude extracts of various Bacteroidetes-derived LPS act as antagonists for TLR4 and to attenuate the production of inflammatory cytokines [10,11]. It emphasizes the need to uncover the structures of various Bacteroidetes lipid A. Early studies indicated that lipid A of *Bacteroides fragilis*, one of the predominant Bacteroidetes in the human gut, consisted of one phosphate group and five acyl chains with various chain lengths [12,13]; however, lipid A structures of other various Bacteroidetes species are unknown. Quantitative and qualitative analyses of lipid A in each bacterium are expected to help us understand the strain-specific phenotypes [11,14]. For this purpose, the development of an analytical method for various lipid A molecular species is essential.

To date, the structure of intact lipid A has been investigated mainly by matrix-assisted laser desorption/ionization (MALDI) mass spectrometry (MS) [15,16,17] and direct infusion-electrospray ionization MS [18,19]. There are a few examples of mass spectrometry coupled with separation methods, such as reverse-phase liquid chromatography (LC), which can be employed for elucidating the structure of intact lipid A [20,21]. The difficulty in the analysis of intact lipid A by LC-MS is due to the diverse physical properties arising from different numbers of acyl chains and phosphate groups [21]. In particular, phosphate groups tend to form chelates with metals in LC columns and stainless-steel piping, hindering the acquisition of sharp chromatographic peaks [22,23]. To overcome this problem, ion-pair reagents have been often used. For example, a method using triethylamine as an ion-pair reagent successfully revealed the heterogeneity of lipid A in *E. coli*, *Salmonella* Adelaide, and *Proteus morganii* [21].

In this study, we developed an analytical system based on LC-quadrupole time-of-flight (QTOF)/MS without ion-pair reagents. The method was validated by analyzing well-characterized *E. coli* lipid A extracts and subsequently applied to lipid A extracts of nine representative Bacteroidetes strains to reveal the structural diversity of lipid A molecular species.

## 2. Results

### 2.1. Development of an Analytical Method for Lipid A

**Figure 1:**
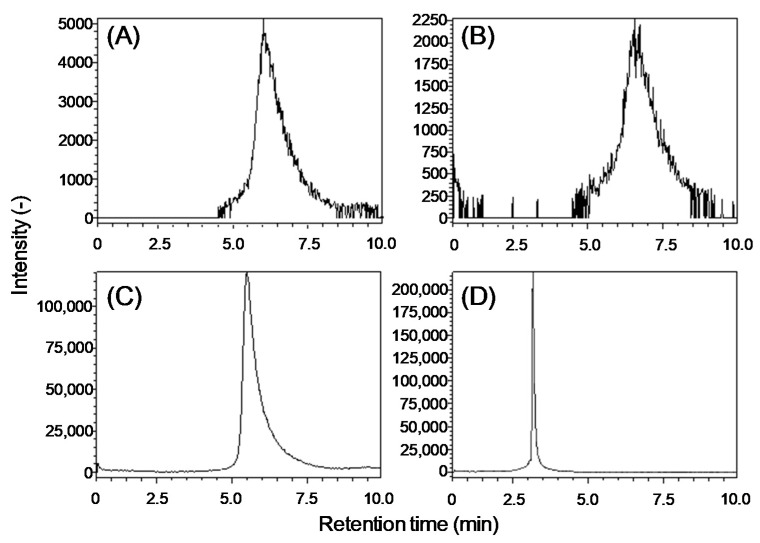
Optimization of reverse-phase LC-MS solvent conditions. Extracted-ion chromatogram of 5 µM of *E. coli* lipid A standard (*m*/*z* = 1796.211). Solvents A and B consisted of a 9:1 (*v*/*v*) methanol:water mixture and 100% 2-propanol, respectively, supplemented with (**A**) 10 mM of ammonium acetate, (**B**) 100 mM of ammonium acetate, (**C**) 10 mM of ammonia, or (**D**) 100 mM of ammonia.

Lipid A molecules contain one or two phosphate groups, which can cause peak tailing due to adsorption to the LC stainless-steel piping. We examined the pH of the mobile phase since pH optimization is critical for obtaining sharp peaks of phosphate-containing compounds [23]. A YMC-Triart C18 metal-free column was employed, which is suitable for a broad range of pH (1–12), and the *E. coli* lipid A standard was analyzed using mobile phases under acidic, neutral, and alkaline conditions (Figure 1). A 9:1 (*v*/*v*) methanol:water mixture and 100% 2-propanol were chosen as the original solvents A and B, respectively. No peaks of lipid A [M−H]^−^ were observed under acidic conditions supplemented with 10 or 100 mM acetic acid (data not shown). Neutral conditions with 10 or 100 mM ammonium acetate showed broad lipid A peaks (Figure 1A,B). An alkaline solvent with 10 mM ammonia dramatically improved the sensitivity, although the peak was still broad (Figure 1C). Increased ammonia concentrations up to 100 mM achieved high sensitivity and a sharp peak simultaneously, but the retention time became shorter (Figure 1D). To increase the retention capacity of lipid A, the water proportion and ammonia concentration in the solvents were further optimized. The retention time increased proportionally with the increase in the percentage of water, and the increased ammonia concentration enhanced the peak height (data not shown). Finally, we optimized the conditions as follows: solvent A, an 8:2 (*v*/*v*) methanol:water mixture supplemented with 200 mM of ammonia; solvent B, 100% 2-propanol supplemented with 200 mM of ammonia. Although alkaline conditions are generally detrimental to a typical C18 column, this highly durable column was reproducible. The limit of detection was 100 nM, as determined through the sequentially diluted standards.

### 2.2. Analysis of Lipid A in E. coli Wild Type and Gene Knockout Strains

**Figure 2:**
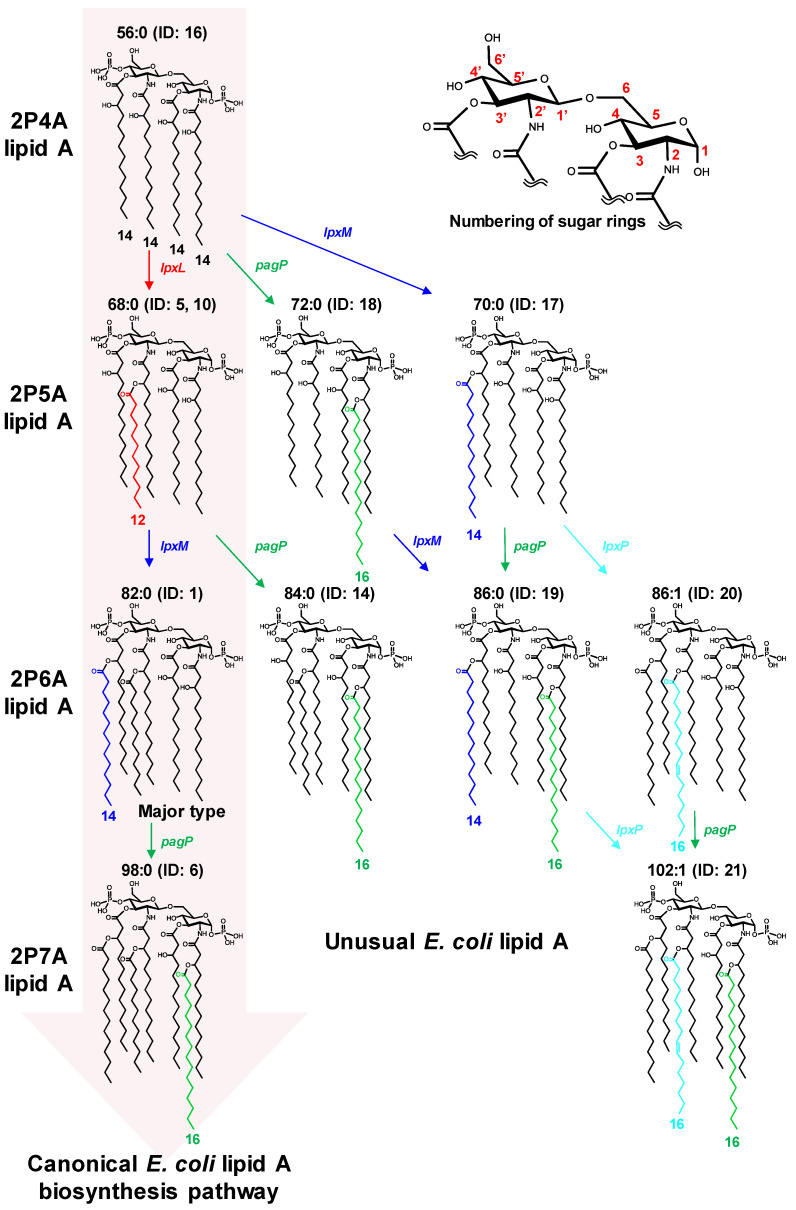
Canonical and proposed metabolic pathways of *E. coli* lipid A. The pink arrow indicates the canonical lipid A biosynthesis pathway in *E. coli*. A moiety of 3-deoxy-d-manno-octulosonic acid (kdo_2_) linked to 6′-O position and the reaction of kdo transferase are not shown. The upper right panel shows the numbering of the sugar rings. Carbon chain lengths are written under the acyl chain. Double bond positions in the structures of ID 20 and 21 were estimated from the literature [28].

**Figure 3:**
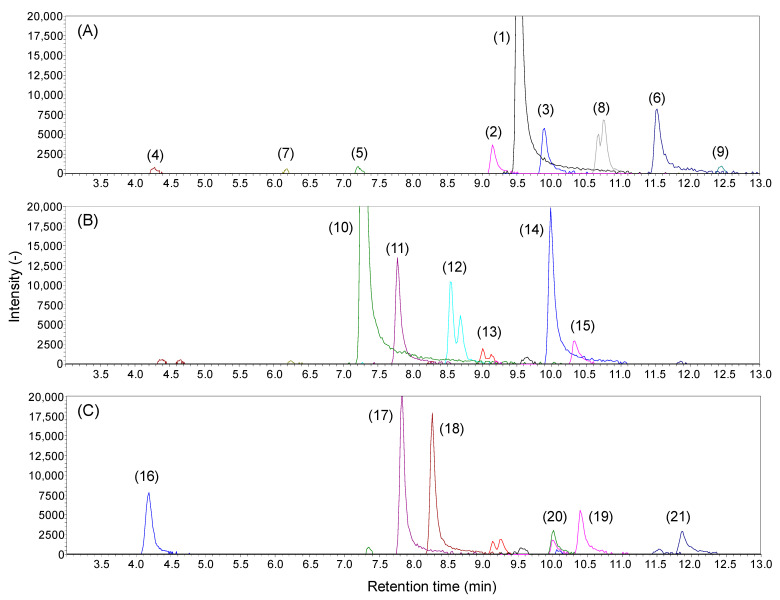
Extracted ion chromatograms of lipid A obtained from *E. coli* (**A**) wild type, and gene knockout strains of (**B**) *lpxM* and (**C**) *lpxL*. Elucidated structures and LC-MS data are summarized in Table 1.

To validate the developed LC-MS method, we analyzed well-characterized lipid A crude extracts from cultured *E. coli* in a data-dependent acquisition mode using LC-QTOF/MS. The structure of the dominant lipid A molecule in *E. coli* is known as diphosphoryl hexa-acylated (2P6A) lipid A consisting of 2-*N*-C14:0(3-OH), 3-*O*-C14:0(3-OH), 2′-*N*-C14:0(3-*O*-C12:0), and 3′-*O*-C14:0(3-*O*-C14:0) on a diphosphoryl diglucosamine backbone (ID: 1 in Figure 2). In addition, some minor molecular species with different numbers of phosphate groups and side chains have been reported [20,21]. In this study, the lipid A structure above is described as 2P6A lipid A 2-*N*-C14:0(3-OH)_3-*O*-C14:0(3-OH)_2′-*N*-C14:0(3-*O*-C12:0)_3′-*O*-C14:0(3-*O*-C14:0), and is abbreviated as 2P6A lipid A 82:0 based on the total number of carbon atoms, and degree of unsaturation in the acyl chains. Numbering of sugar rings is described in Figure 2. Crude LPS was extracted from aerobically cultured *E. coli* BW25113 cells using the TRI Reagent method [24]. Lipid A was isolated from the polysaccharide part via mild acid hydrolysis [24]. The LC-QTOF/MS raw data were processed using MS-DIAL software [25]. The total fatty acid chain length, unsaturation degree, and amount of oxygen in hydroxyl fatty acids were estimated from the measured exact *m/z* (Δ < 5 ppm). The structure moiety was evaluated from MS/MS spectra containing phosphate-related peaks (*m/z* = 78.958 or 96.969). The annotated peaks corresponding to the ions of [M−H]^−^ or [M−2H]^2−^ are shown in Table 1. A large peak corresponding to 2P6A lipid A 82:0 (*m/z* = 1796.203) was observed (ID: 1 in Figure 3A) with some small peaks in the LC-MS chromatogram (ID: 2–9 in Figure 3A). As expected, the MS/MS spectrum of the largest peak matched that of the known *E. coli* lipid A molecule peak (Table 1 and Figure S1A). Two additional peaks were observed near the main peak (ID: 2 and 3). Following the previous fragmentation rules, those peaks were identified as 2P6A lipid A 84:0 and 80:0, in which the side chains of 2′-*N*-acyl and 3′-*O*-acyl were swapped from C12:0 to C14:0 and from C14:0 to C12:0, respectively (Table 1 and Figure S1B,C). Further analysis identified lipid A molecules with different numbers of acyl chains, such as 2P tetra-acylated (4A) lipid A 54:0 (ID: 4), 2P penta-acylated (5A) lipid A 68:0 (ID: 5), and 2P hepta-acylated (7A) lipid A 98:0 (ID: 6) (Figure S1D–F). Monophosphoryl (1P) lipid A variants, estimated as 1P4A lipid A 54:0 (ID: 7), 1P6A lipid A 84:0 (ID: 8), and 1P7A lipid A 98:0 (ID: 9) were also observed. Two separated peaks of 1P lipid A molecules may correspond to lipid A with different phosphorylation positions (1 or 4′ on the diglucosamine backbone, Figure S1G). Elucidated structures of minor lipid A species were consistent with those reported in previous studies [20,21].

Next, we analyzed the *E. coli lpxM* gene knockout strain to test whether the developed method could determine the expected changes in lipid A molecular species. LpxM is an enzyme that forms the sixth *O*-C14:0 side chain on the 3′-*O*-C14:0 (3-OH) of 2P5A lipid A 68:0 in the canonical *E. coli* lipid A biosynthesis pathway (Figure 2). The LC-MS chromatogram of the *lpxM* gene knockout strain revealed the absence of 2P6A lipid A 82:0 and accumulation of 2P5A lipid 68:0 (ID: 10 in Figure 3B and Figure S1H), demonstrating that our method can clearly capture the genetic perturbation of the lipid A biosynthetic pathway in *E. coli*.

Interestingly, we found more minor peaks, most of which were not detected in the *E. coli* wild-type strain. For example, 2P5A lipid A 70:0 (ID: 11), where the side chain of 2′-*N*-acyl of 2P5A lipid A 68:0 was replaced with C14:0 instead of C12:0, and the monophosphorylated forms, 1P5A lipid A 68:0 (ID: 12) and 1P5A lipid A 70:0 (ID: 13), were identified (Figure S1I–L). MS/MS spectra obtained from the left and right peaks of 1P5A lipid A 68:0 (ID: 12) showed a characteristic fragmentation pattern which could estimate the phosphate positions on diglucosamine backbone. The MS/MS spectrum of the right peak (Figure S1K) was similar to the known fragmentation of 4′-monophosphorylated lipid A [15]. In contrast, the MS/MS spectrum of the left peak contained unique ions with *m/z* = 591.270 and 633.281 (Figure S1J). The fragment ions could be estimated as the cleavage of amide, and C-C bonds next to the 3-hydroxy group, respectively, of the 2-*N*-acyl chain in 1-monophosporyl lipid A (cleavage of (f) and (g) in Figure S1J). Despite the deletion of the sixth acyl chain-introducing enzyme lpxM, several hexa-acylated lipid A forms, such as 2P6A lipid A 84:0 (ID: 14) and 2P6A lipid A 86:0 (ID: 15) were also detected (Figure S1M,N). Structure estimation demonstrated that the side chain on 2′-*N*-acyl was absent, which was consistent with the *lpxM* knockout, but hydroxy fatty acids linked to the 2-*N* position were further acylated by C16:0. Acylation at this position is catalyzed by pagP in the conversion of 2P6A to 2P7A lipid A [14,26]. These results indicate that pagP also acts on 2P5A lipid A (Figure 2).

Analysis of the *lpxL* knockout strain, which lacked an enzyme to introduce the fifth acyl chain into 2P4A lipid A in the *E. coli* lipid A pathway (Figure 2), also showed a unique lipid A profile (Figure 3C). The characteristic presence and absence, respectively, of 2P4A lipid A 56:0 (ID: 16, Figure S1P) and 2P5A lipid A 68:0 indicated the inactivation of the lpxL reaction [27]. A remarkable accumulation of 2P5A lipid A 70:0 (ID: 17) and 72:0 (ID: 18) was found in the *lpxL* knockout strain (Figure S1Q,R). MS/MS spectra assignments demonstrated that the hydroxyl groups on the 3′-*O*- and 2-*N*-main chains were acylated by C14:0 and C16:0, respectively, suggesting that lpxM and pagP can acylate 2P4A lipid A as a substrate (Figure 2). 2P6A lipid A 86:0 (ID:19) was estimated as the product of sequential reaction of lpxM and pagP (Figure 2). Although the peak height was moderate, we also found unique unsaturated 2P6A lipid A 86:1 (ID: 20, Figure S1T) and 2P7A lipid A 102:1 (ID: 21). It was estimated that the side chains on 2′-*N*-acyl in these structures were of the C16:1 type. The acylation of C16:1 to this position is catalyzed by lpxP (Figure 2), whose expression is induced by low temperatures [28]. This observation suggests that lpxP expression could be induced to compensate for the *lpxL* gene knockout, even at the optimum temperature [27]. These unique insights into the heterogeneity of *E. coli* lipid A in gene knockout strains could be obtained by employing the current LC separation technique coupled to QTOF/MS.

### 2.3. Analysis of Lipid A in Bacteroidetes Bacteria

**Figure 4:**
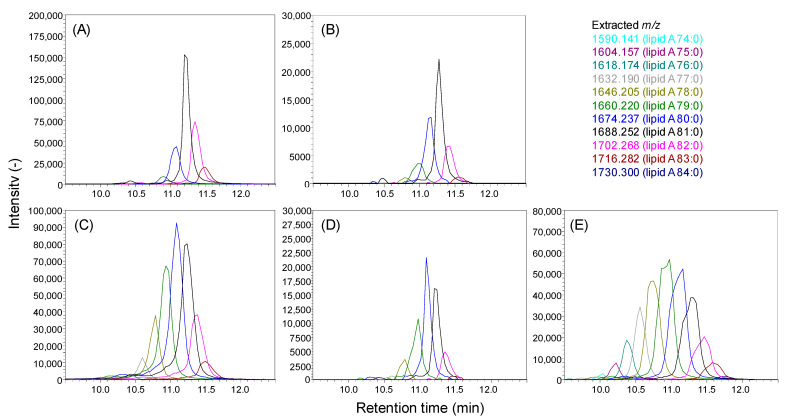
Lipid A in Bacteroidetes strains. Extracted-ion chromatograms of lipid A obtained from (**A**) *B. fragilis*, (**B**) *B. thetaiotaomicron*, (**C**) *B. vulgatus*, (**D**) *O. laneus*, and (**E**) *P. copri.* MS1 spectra of lipid A extracts of all Bacteroidetes were presented in Figure S4.

**Figure 5:**
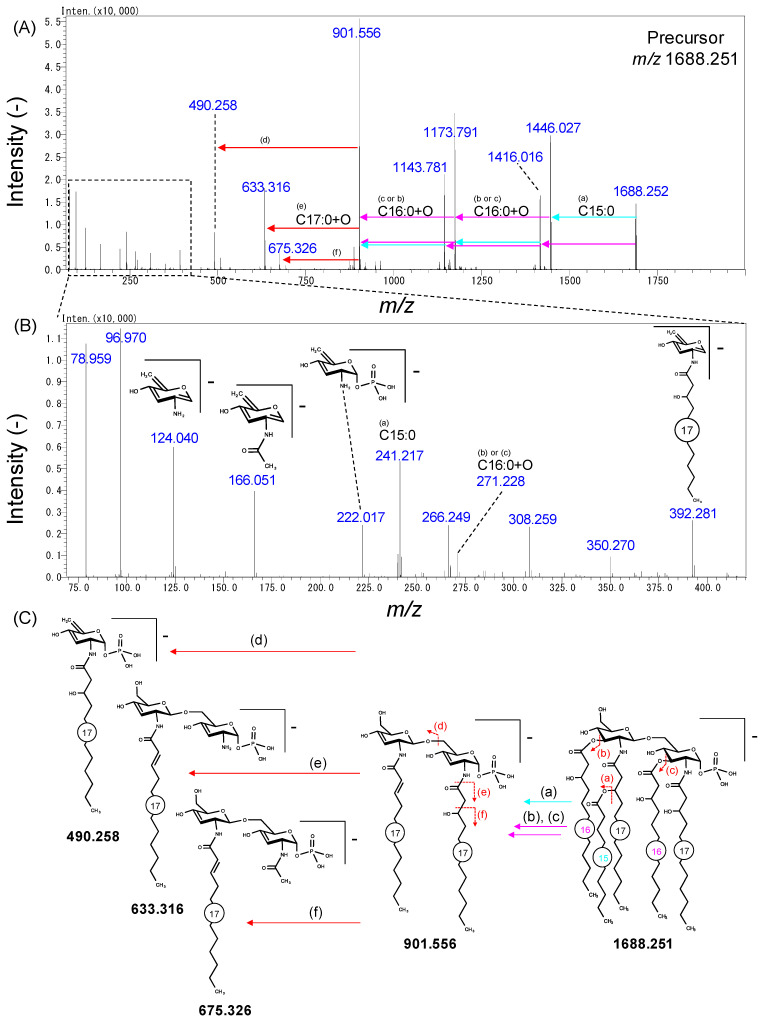
Structural estimation of *B. fragilis* lipid A. (**A**) MS/MS spectrum of 1688.251. Monoisotopic *m/z* are given. Arrows indicate the neutral losses. (**B**) Zoomed spectrum of the dashed region shown in (**A**). Estimated fragment ion structures are presented. (**C**) A proposed fragmentation scheme of 1P5A lipid A 81:0. The numbers in circles on acyl chains indicate the chain lengths. (**a**–**f**) correspond to the cleavage sites in the chemical structure of (**C**).

Next, we applied the developed method in the analysis of lipid A in Bacteroidetes strains. Nine phylogenetically diverse strains, *B. fragilis*, *B. uniformis*, *B. vulgatus*, *B. intestinalis*, *B. thetaiotaomicron*, *Parabacteroides johnsonii*, *Odoribacter laneus*, *Prevotella copri*, and *Paraprevotella xylaniphila*, were anaerobically cultured in modified Gifu anaerobic medium (mGAM). LC-QTOF/MS analysis of *B. fragilis* lipid A showed a variety of peaks with an *m/z* difference of 14.02 (Figure 4A), the largest of which was the peak with an *m/z* = 1688.252 (lipid A 81:0). The MS/MS spectra of that peak showed phosphoglucosamine-derived ions (*m/z* = 124.040, 166.051, and 222.017, Figure 5B), suggesting that *B. fragilis* lipid A contains a phosphoglucosamine backbone. We found that an ion with an *m/z* = 901.556 was generated by neutral losses corresponding to one C15:0 and two C16:0+O (Figure 5A), suggesting that these fatty acids were ester-linked to the hydroxyl group of one of the main fatty acids and the diglucosamine backbone, respectively (Figure 5C, neutral losses of (a), (b) and (c)). The ion was further fragmented to generate ions with *m/z* = 490.258, 633.313, and 675.326, matching the cleavage of (d), (e), and (f), respectively (Figure 5A,C). The 490.258 ion was identified as a diagnostic ion to determine the 2-*N*-acyl chain length (Figure 5C). From the ion with an *m/z* = 633.313, we estimated a chain length of C17:1 for the 2′-*N*-acyl chain. The mono-unsaturation was interpreted as an evidence of dehydration caused by the neutral loss of side-chain fatty acids (Figure 5C). The ion with an *m/z* = 675.326 was interpreted as an indication that the hydroxyl group of the fatty acid was in the beta position (Figure 5C). This fragmentation pattern was similar to that of 1-monophosphoryl 5A lipid A 68:0 found in *E. coli* (Figure S1J), validating that the 1-*O* position was phosphorylated in *B. fragilis* lipid A. Since we could not find any fragment ions to differentiate the ester-linked fatty acid position, we assumed that the main chain was C16:0(3-OH) based on previous knowledge [12,13]. The remaining C15:0 was likely esterified to the hydroxyl group on the 2′-*N*-acyl chain.

**Figure 6:**
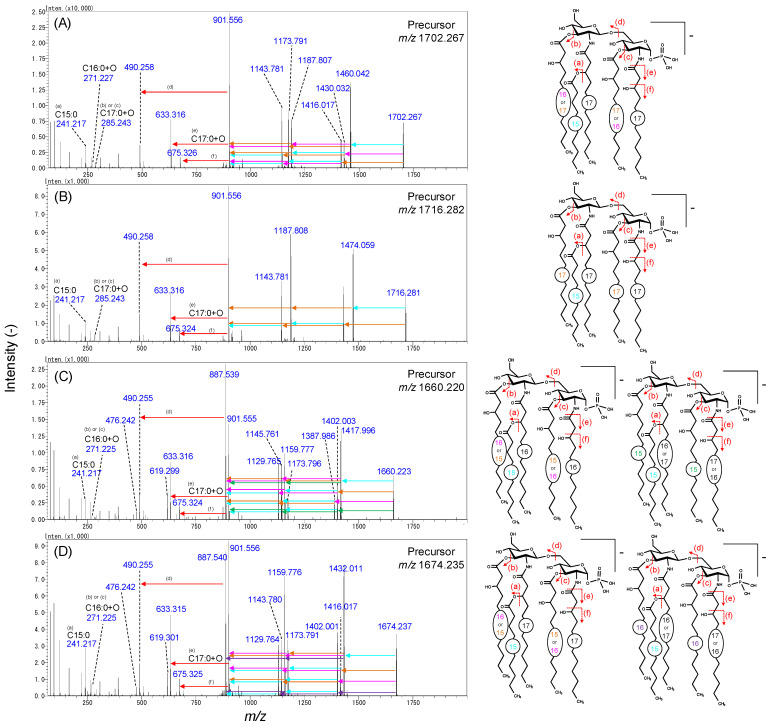
MS/MS spectra and proposed structural assignments of *B. fragilis* lipid A. (**A**) 1P5A lipid A 82:0, (**B**) 1P5A lipid A 83:0, (**C**) 1P5A lipid A 79:0, and (**D**) 1P5A lipid A 80:0. The arrows of each color indicate the neutral loss of the acyl chain of the same color. Monoisotopic *m/z* are indicated. (**a**–**f**) correspond to the cleavage sites in the chemical structure on the right side.

Figure 4A also shows other peaks corresponding to lipid A 79:0, 80:0, 82:0, and 83:0. The structure of lipid A 80:0, the second largest peak in Figure 4A, was estimated considering that either the 3-*O*-acyl- or 3′-*O*-acyl chain had been replaced from C16:0+O to C17:0+O, although the side chain was conserved as C15:0 (Figure 6A). Similarly, the ion with the largest *m/z* = 1716.282 was assumed to be the molecule with all main hydroxy fatty acid lengths equal to 17 (Figure 6B). Next, the structures with smaller *m/z* than 1P5A lipid A 81:0 were elucidated (Figure 6C,D). The MS/MS spectra exhibited neutral loss corresponding to C15:0+O, C16:0+O, C17:0+O, and C15:0. Two diagnostic ions (*m/z* = 490.255 and 476.242) determining the 2-*N*-acyl chain type indicated that the chain length was either C16:0+O or C17:0+O. Based on these fragmentation patterns, we hypothesized that the precursor ion was a mixture of four isomers, as shown in Figure 6C. A similar interpretation was applied for the ion at *m/z* = 1674.235 (Figure 6D). These results highlighted the heterogeneity of lipid A in the Bacteroidetes strains owing to the diversity in the main chain length, while the side chain was relatively conserved as C15:0.

Other Bacteroidetes lipid A structures were also investigated. As shown in Figure 4B–E, a strain-specific pattern of lipid A species was observed. The extracted-ion chromatogram of *B. thetaiotaomicron* showed that lipid A 81:0 was the predominant species, while the second one was lipid A 80:0 (Figure 4B). For *B. vulgatus*, and *O. laneus*, lipid A 80:0 was the most abundant species (Figure 4C,D). *P. copri* lipid A showed a highly heterogeneous distribution around the peak of lipid A 79:0, including a relatively lower total acyl chain length (C74 to C76). The structures of lipid A 81:0 from *B. thetaiotaomicron*, *B. intestinalis*, *B. uniformis*, and *B. vulgatus* were almost identical to those of *B.* *fragilis* lipid A 2-*N*-C17:0(3-OH)_3-*O*-C16:0(3-OH)_2′-*N*-C17:0(3-O(C15:0))_3′-*O*-C16:0(3-OH), while those in *O. laneus* and *P. copri* were different (Figure S2). The acyl chain of both or one of the 2- and 2′-*N* position in the major *O. laneus* lipid A 80:0 was replaced from C17:0+O to C16:0+O (Figure S2G). The *P. copri* lipid A structure was even more diverse: C14:0 and C15:0 appeared as a side chain, and the main acyl chains also exhibited a certain diversity (Figure S2H). The side chain diversity was slightly found in *B. vulgatus* (Figure S2F). The reason why each peak appeared broader than the other in *P. copri* and *B. vulgatus* was probably the co-elution of isomers which had a different side chain (Figure 4C,E). The MS/MS spectra and estimated structures of major lipid A in *B. vulgatus* (80:0), *O. laneus* (80:0), and *P. copri* (79:0) are presented in Figure S3. The major *P. copri* lipid A also had a C13:0 side chain (Figure S3C). These observations highlighted the importance of determining the lipid A acyl chain property in each strain.

**Figure 7:**
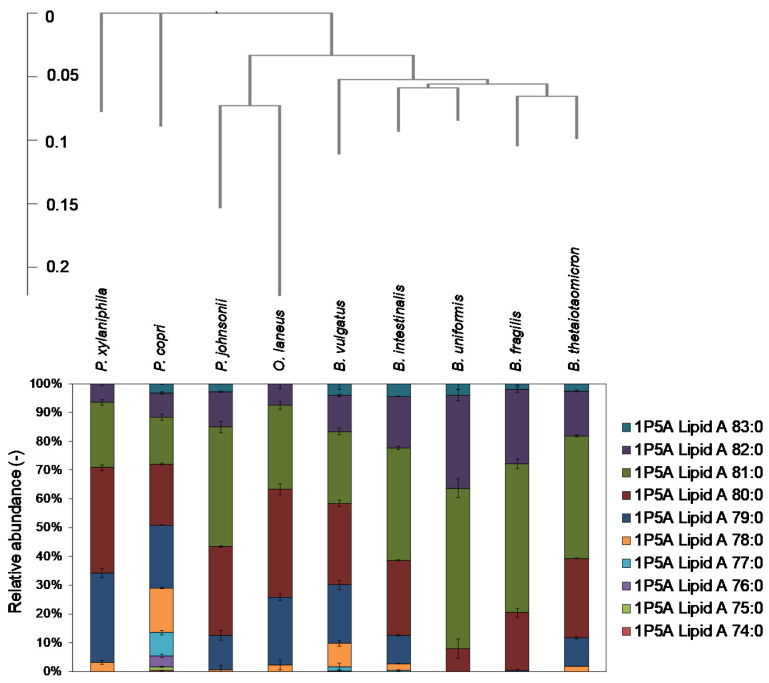
Relative peak areas of 1P5A lipid A molecular species in Bacteroidetes strains. The phylogenic tree was created based on the 16S ribosomal RNA sequences.

Finally, the relative peak areas of 1P5A lipid A in Bacteroidetes strains are shown in Figure 7. These results suggest that 1P5A lipid A 81:0 is a major molecule in *B. fragilis* and genetically similar Bacteroidetes species (39.0–55.7%), while lipid A types with relatively lower total chain length (<C81) are enriched in distantly related species (Figure 7). The peak numbers and distributions of 1P5A lipid A molecular species were almost identical in a preliminary experiment performed on different days under the same culture condition (data not shown). In summary, the heterogeneity of lipid A molecules in Bacteroidetes strains is related to the phylogenetic similarity of strains.

## 3. Discussion

To develop an optimized reverse-phase LC method coupled with QTOF/MS for lipid A analysis, we investigated the pH conditions of the mobile phase and observed a sharp lipid A peak in alkaline conditions (Figure 1D). In general, acidic conditions are employed for the separation of anionic compounds in a C18 column to improve their retention capacity. However, in our study, no lipid A peaks were observed in the acidic solvents, whereas the basic solvent dramatically improved the sensitivity and peak shape. These results indicate that lipid A formed a chelate with the stainless-steel surface of the injector, fitting, and electrode despite employing a metal-free column, and the effect was reduced using a basic solvent. Additionally, basic solvents could contribute to achieving high sensitivity toward signals derived from phosphate compounds since the anionic state in the basic solvent enhances the negative-mode ionization efficiently. In this study, we did not discuss the intensity values themselves since the extraction efficiency of LPS and the hydrolysis efficiency of polysaccharides were not corrected. Spiking fully ^13^C-labeled bacterial cells prepared in ^13^C-substrate would cancel and minimize the effects, resulting in a more reproducible data.

An analysis of wild-type *E. coli* showed heterogeneous profiles of lipid A molecules (Figure 3A, Table 1). Furthermore, *E. coli* gene knockout strains showed a series of unusual lipid A species that were not observed in the wild type (Figure 3B,C, Table 1). The presence of such abnormal lipid A types in these strains was partially indicated in a previous study, although the structural details were not clear [27]. Structural evaluation of the MS/MS spectra allowed for elucidation of the unique lipid A side chain properties and showed that the substitution position was consistent with the known acylation sites by pagP, lpxP, and lpxM, although their substrates were not canonical structures (Figure 2, Table 1). This indicates that the substrate specificity of these enzymes is not strictly dependent on the number of acyl groups in the substrate.

The analysis of Bacteroidetes strains showed that the most abundant lipid A type in *B. fragilis* was lipid A 81:0 (Figure 4A). Considering the biosynthetic pathway, hydroxy fatty acids are probably linked to the 3-*O*- and 3′-*O* positions of the diglucosamine backbone [8,9]; therefore, the structure was annotated as 1P5A lipid A 2-*N*-C17:0(3-OH)_3-*O*-C16:0(3-OH)_2′-*N*-C17:0(3-O(C15:0))_3′-*O*-C16:0(3-OH) (Figure 5). Although an early study demonstrated the structure of *B. fragilis* lipid A was 1P5A lipid A 79:0; 2-*N*-C16:0(3-OH)_3-*O*-C15:0(3-OH)_2′-*N*-C17:0(3-O(C15:0))_3′-*O*-C16:0(3-OH) [12], recent studies showed that *m/z* of major molecular species were larger than 1P5A lipid A 79:0 [10,29], which is consistent with our results (Figure 4). Structural assessment of lipid A types with different *m/z* revealed a diversity in the main chain length with a combination of C15:0+O, C16:0+O, and C17:0+O, while the side chain length was constant at C15:0 (Figure 6). This is in contrast with the diversity observed in the *E. coli* lipid A side chains. One of the reasons for this difference is possibly the number of acyltransferases in each organism. *E. coli* has four enzymes, lpxL, lpxM, lpxP, and pagP, but only lpxL is annotated in Bacteroidetes [30]. However, it is unclear why the diversity of the main chains is observed in Bacteroidetes but not in *E. coli*. This may be related to the differences in substrate specificity of the main chain-introducing enzymes (lpxA and lpxD) or fatty acid availability between *E. coli* and Bacteroidetes strains.

The 1P5A lipid A distribution was different among the nine Bacteroidetes strains (Figure 4). *B. intestinalis*, *B. uniformis*, and *B. thetaiotaomicron*, which are genetically similar to *B. fragilis*, had 1P5A lipid A 81:0 as the predominant species (Figure 7). In contrast, the second most common lipid A in these strains was 1P5A lipid A 80:0 for *B. fragilis* and *B. uniformis*, and 1P5A lipid A 82:0 for *B. intestinalis* and *B. thetaiotaomicron* (Figure 7). In species that are phylogenetically distant from these four strains, the major lipid A was 1P5A 80:0, with a trend toward more lipid A types with fewer carbons, except for *P. johnsonii* (Figure 7). These results suggest that lipid A molecular profiles can be used as a taxonomic index and detection marker for bacteria. In fact, research on the detection of drug-resistant and pathogenic bacteria focusing on lipid A has recently emerged [31,32,33], and it is important to determine the structural diversity of lipid A for each bacterial strain and related phenotypes. The present study will contribute to deciphering the complex and heterogeneous landscape of lipid A in bacteria.

## 4. Materials and Methods

### 4.1. Strains and Cultures

*E. coli* BW25113 and their single gene knockout strains (*lpxL* and *lpxM*) were obtained from the National Institute of Genetics (Mishima, Japan) [34]. *E. coli* strains were aerobically cultured on Luria–Bertani (LB) agar (Merck, Darmstadt, Germany) plates at 37 °C. A single colony was inoculated into 5 mL of LB liquid medium and aerobically cultured overnight in a BR-43 FL shaker (TAITEC, Tokyo, Japan) at 37 °C with 120 rpm reciprocal shaking. *B. fragilis* (JCM11019), *B. thetaiotaomicron* (JCM5827), *B. vulgatus* (JCM5826), *B. intestinalis* (JCM13265), *B. uniformis* (JCM5826), *P. johnsonii* (JCM13406), *P. copri* (JCM13464), *P. xylaniphila* (JCM14860), and *O. laneus* (JCM16069) were obtained from the Riken Bioresource Research Center (Tsukuba, Japan). The intestinal bacteria were cultured in mGAM (Nissui Pharmaceutical, Tokyo, Japan) agar plates. Single colonies were inoculated into 10 mL of mGAM and cultured overnight at 37 °C in a type A vinyl anaerobic chamber operated in 10% H_2_, 10% CO_2_, 80% N_2_, O_2_ < 5 ppm (Coy Laboratory Products, Grass Lake, MI, USA). The culture broth was centrifuged at 2900× *g* for 20 min at 4 °C. The cell pellets were washed twice with 10 mL of phosphate-buffered saline. Cell pellets were stored in a −80 °C freezer until analysis.

### 4.2. Lipid A Extraction and Analysis

Total lipopolysaccharides were isolated using the TRI Reagent method [21]. Polysaccharides were removed by weak acid hydrolysis, as described previously [21]. Lipid A was analyzed using UHPLC (Nexera X2, Shimadzu, Kyoto, Japan) coupled with a QTOF/MS (LCMS-9030, Shimadzu, Kyoto, Japan). Lipids were separated on a YMC-Triart C18 column (50 × 2.0 mm, 1.9 µm; YMC, Kyoto, Japan). The column was maintained at 45 °C at a flow rate of 0.3 mL/min. The mobile phases consisted of (A) 8:2 (*v*/*v*) methanol:water and (B) 100% 2-isopropanol. Both solvents were supplemented with 1.5 mL of a 25% ammonium hydroxide solution per 100 mL solvents (200 mM in final). The separation was conducted under the following gradient: 0 min 0% (B), 2 min 0% (B), 17 min 95% (B), 17.1 min 0% (B), and 20 min 0% (B). Data-dependent MS/MS acquisition was performed. The QTOF/MS settings were as follows: MS1 scan range, 650–2000; MS2 scan range, 70–2000; collision energy, 85; collision energy spread, 20; Q1 resolution, low; MS1 threshold, high; MS2 threshold, 1200; ionization mode, electrospray ionization; nebulizer gas flow, 2.0 L/min; heating gas flow, 10 L/min; interface temperature, 300 °C; drying gas flow, 10 L/min; desolvent line temperature, 300 °C; heat block temperature, 400 °C; DDA event number, 10; precursor search *m/z* range, 650–2000. Data were analyzed using LabSolutions software v. 5.99 SP1 and MS-DIAL [22].

### 4.3. Creation of Phylogenic Tree

Phylogenic tree was created based on the 16S ribosomal RNA sequences using the FastME program [35] implemented in NGPhylogeny.fr [36]. 16S ribosomal RNA sequences were obtained from the webpage of the National Center for Biotechnology Information.

## Figures and Tables

**Table 1:** Estimated structure moiety of *E. coli* lipid A.

Peak ID	Retention Time (min)	Measured *m/z*	Ion Types	Compounds	Class	Acyl Property			
						C3′	C2′	C3	C2
7	6.2	1279.848	[M−H]^−^	Lipid A 54:0	1P4A *^1^	N.D. *^2^			
4	4.3	1359.813	[M−H]^−^	Lipid A 54:0	2P4A	OH	C14:0(O-C12:0)	C14:0(OH)	C14:0(OH)
16	4.2	1403.841	[M−H]^−^	Lipid A 56:0	2P4A	C14:0(OH)	C14:0(OH)	C14:0(OH)	C14:0(OH)
12	8.6 and 8.7	1506.041	[M−H]^−^	Lipid A 68:0	1P5A	C14:0(OH)	C14:0(O-C12:0)	C14:0(OH)	C14:0(OH)
13	9.0 and 9.2	1534.072	[M−H]^−^	Lipid A 70:0	1P5A	C14:0(OH)	C14:0(O-C14:0)	C14:0(OH)	C14:0(OH)
5 and 10	7.3	1586.004	[M−H]^−^	Lipid A 68:0	2P5A	C14:0(OH)	C14:0(O-C12:0)	C14:0(OH)	C14:0(OH)
11	7.8	1614.038	[M−H]^−^	Lipid A 70:0	2P5A	C14:0(OH)	C14:0(O-C14:0)	C14:0(OH)	C14:0(OH)
17	7.8	1614.038	[M−H]^−^	Lipid A 70:0	2P5A	C14:0(O-C14:0)	C14:0(OH)	C14:0(OH)	C14:0(OH)
18	8.3	1642.067	[M−H]^−^	Lipid A 72:0	2P5A	C14:0(OH)	C14:0(OH)	C14:0(OH)	C14:0(O-C16:0)
8	10.7 and 10.8	1716.237	[M−H]^−^	Lipid A 82:0	1P6A	C14:0(O-C14:0)	C14:0(O-C12:0)	C14:0(OH)	C14:0(OH)
2	9.2	1768.171	[M−H]^−^	Lipid A 80:0	2P6A	C14:0(O-C12:0)	C14:0(O-C12:0)	C14:0(OH)	C14:0(OH)
1	9.5	1796.203	[M−H]^−^	Lipid A 82:0	2P6A	C14:0(O-C14:0)	C14:0(O-C12:0)	C14:0(OH)	C14:0(OH)
3	9.9	1824.231	[M−H]^−^	Lipid A 84:0	2P6A	C14:0(O-C14:0)	C14:0(O-C14:0)	C14:0(OH)	C14:0(OH)
14	10.1	1824.231	[M−H]^−^	Lipid A 84:0	2P6A	C14:0(OH)	C14:0(O-C12:0)	C14:0(OH)	C14:0(O-C16:0)
20	10.0	1850.250	[M−H]^−^	Lipid A 86:1	2P6A	C14:0(O-C14:0)	C14:0(O-C16:1)	C14:0(OH)	C14:0(OH)
15	10.4	1852.263	[M−H]^−^	Lipid A 86:0	2P6A	C14:0(OH)	C14:0(O-C14:0)	C14:0(OH)	C14:0(O-C16:0)
19	10.4	1852.263	[M−H]^−^	Lipid A 86:0	2P6A	C14:0(O-C14:0)	C14:0(OH)	C14:0(OH)	C14:0(O-C16:0)
9	12.4	1954.465	[M−H]^−^	Lipid A 98:0	1P7A *^1^	N.D. *^2^			
6	11.5	1016.712	[M−2H]^2−^	Lipid A 98:0	2P7A	N.D. *^3^			
21	11.9	1043.736	[M−2H]^2−^	Lipid A 102:1	2P7A *^4^	N.D. *^2^			

N.D., Not determined. *^1^ Estimated from previous study [21]. *^2^ MS/MS spectra were not investigated due to the low intensity. *^3^ Only acyl chain composition was resolved. *^4^ Estimated from the sequential reaction of lpxP and pagP to lipid A 70:0 (ID: 17).

## Data Availability

The data presented in this study are available in supplementary data.

## Supplementary Materials

The following are available online at https://www.mdpi.com/article/10.3390/metabo11040197/s1, Figure S1. MS/MS spectra of diverse molecular species present of *E. coli* lipid A, Figure S2. MS/MS spectra of lipid A from Bacteroidetes strains, Figure S3. MS/MS spectra of dominant lipid A species in each Bacteroidetes strain, Figure S4. MS1 spectra of lipid A extracts in each Bacteroidetes strain.
