# Supplementary material for: Analyses of Lipid A Diversity in Gram-Negative Intestinal Bacteria Using Liquid Chromatography–Quadrupole Time-of-Flight Mass Spectrometry
Source: Metabolites. 2021 Mar 26;11(4):197. doi: 10.3390/metabo11040197 (PMC8065654; doi:10.3390/metabo11040197)
Supplement: Supplementary file 1 [file metabolites-11-00197-s001.pdf]

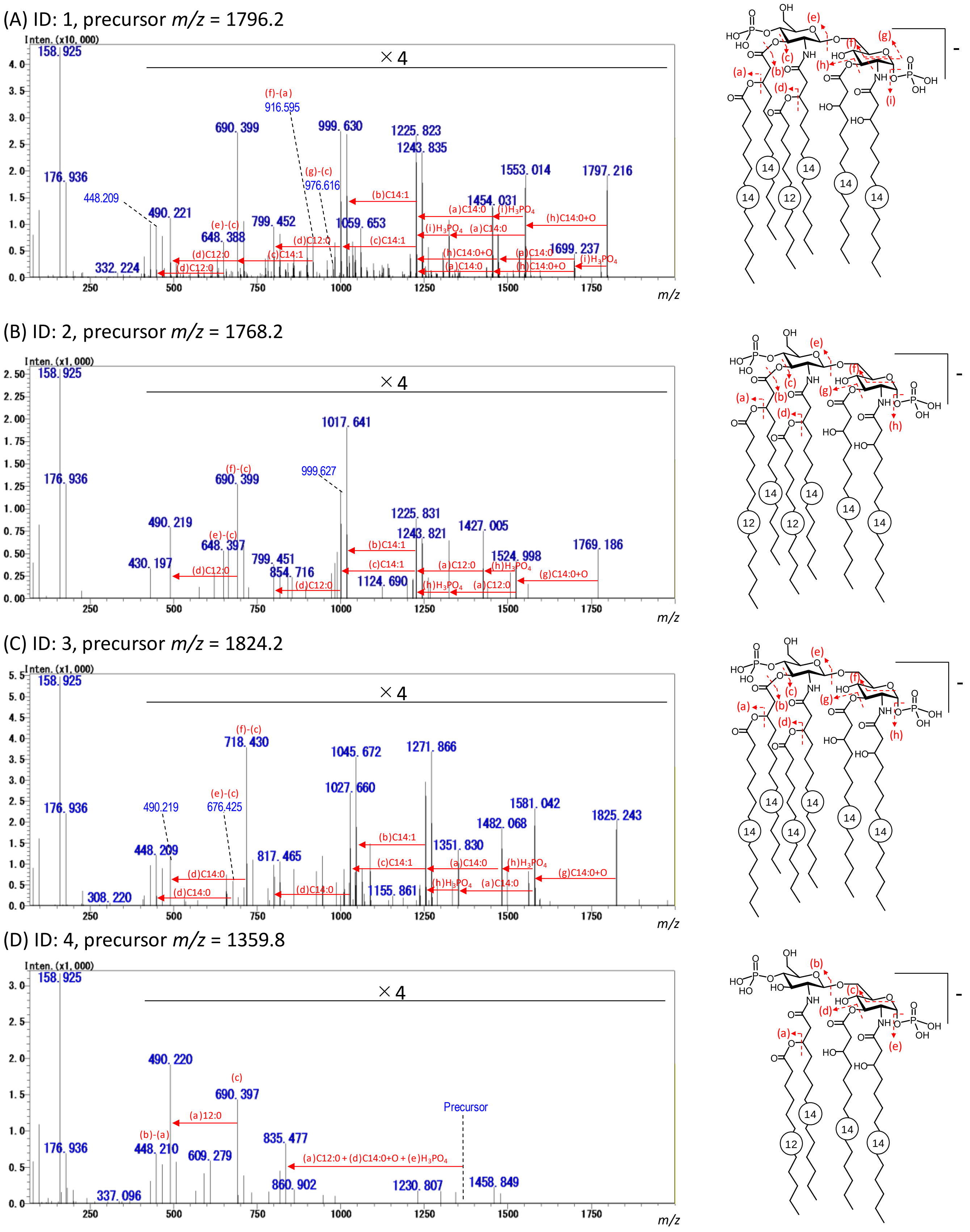

Figure S1. MS/MS spectra of diverse molecular species present in *E. coli* lipid A.

(E) ID: 5, precursor *m/z* = 1586.0

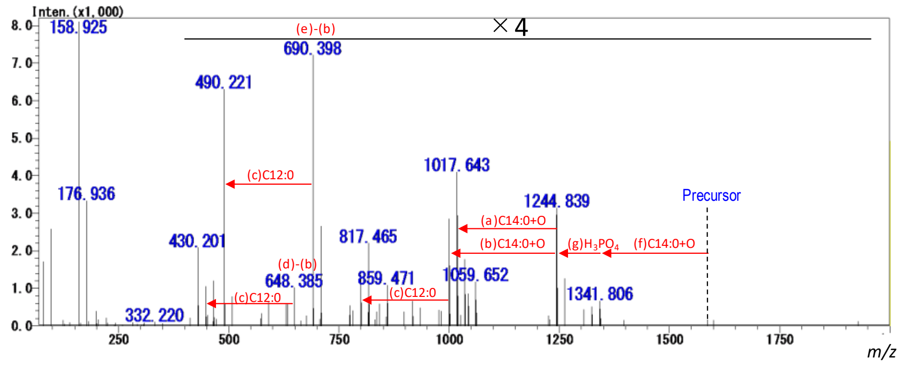

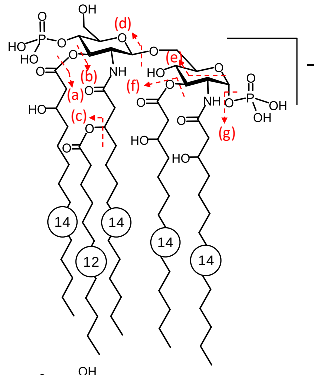

(F) ID: 6, precursor *m/z* = 1016.7

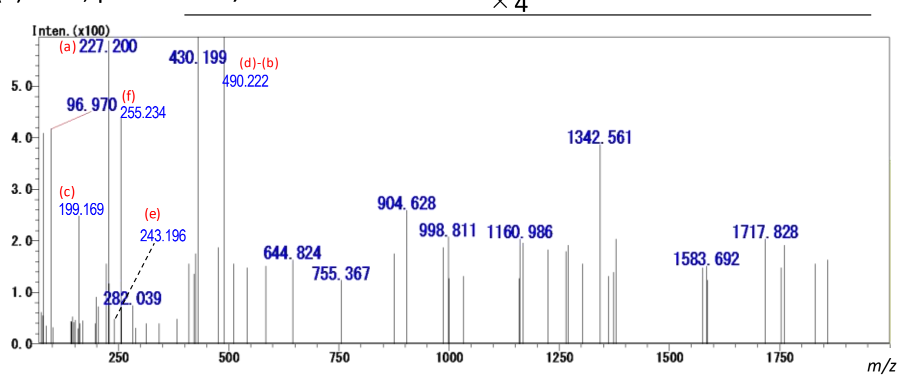

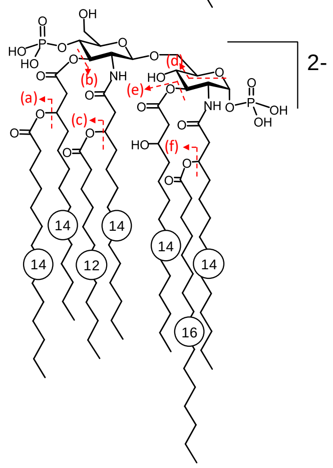

(G) ID: 8, average of left and right peaks, precursor *m/z* = 1716.2

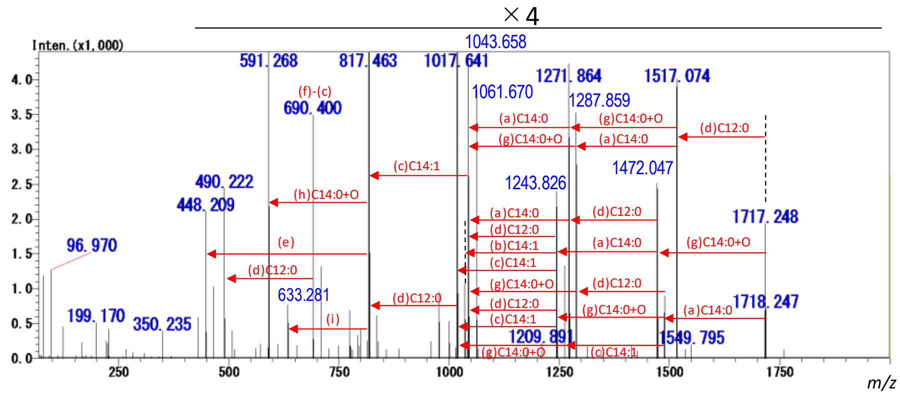

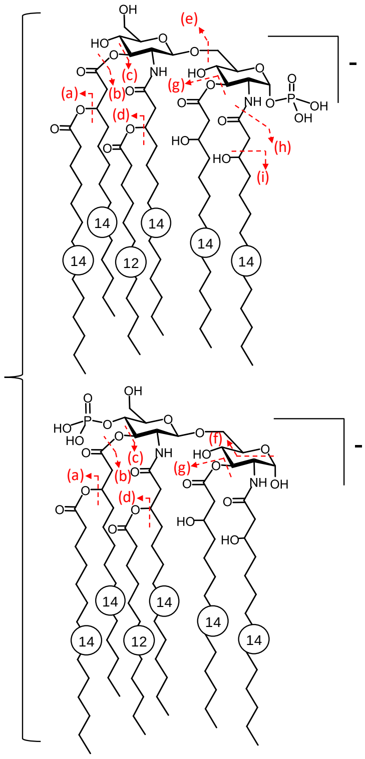

Figure S1. (Continued) MS/MS spectra of diverse molecular species present in *E. coli* lipid A.

(H) ID: 10, precursor m/z = 1586.0

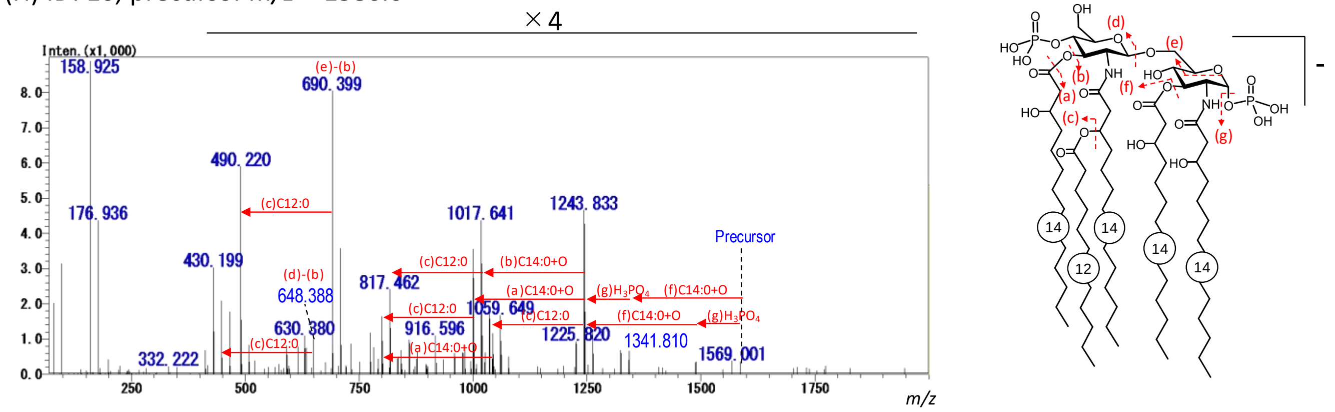

(I) ID: 11, precursor m/z = 1614.0

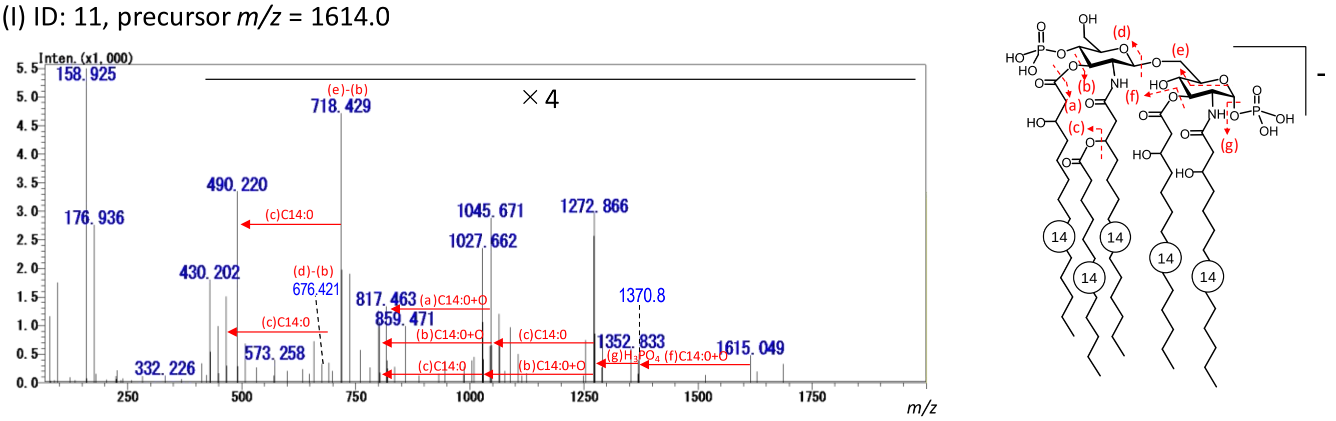

(J) ID: 12 left peak, precursor m/z = 1506.0

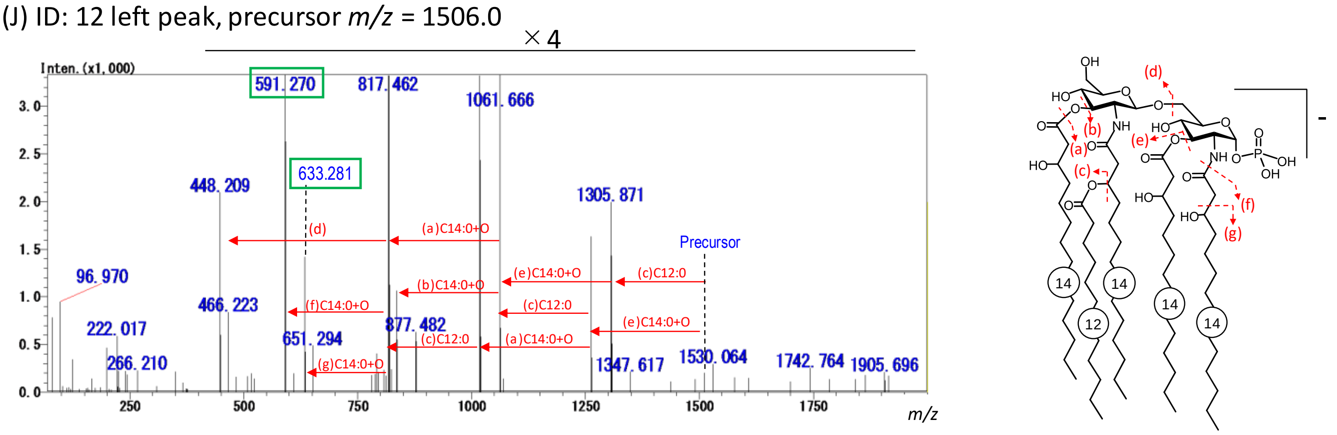

(K) ID: 12 right peak, precursor m/z = 1506.0

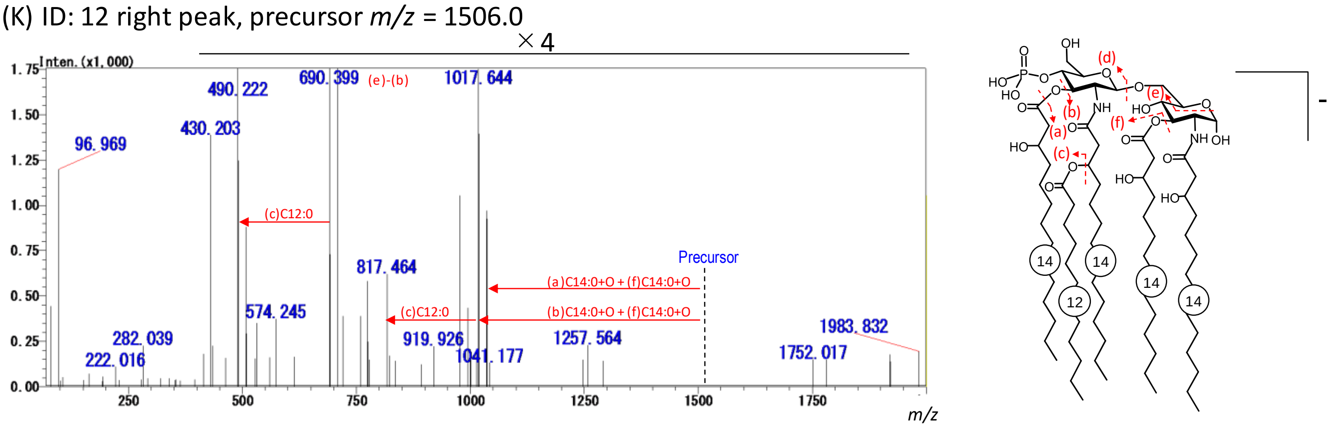

Figure S1. (Continued) MS/MS spectra of diverse molecular species present in *E. coli* lipid A.

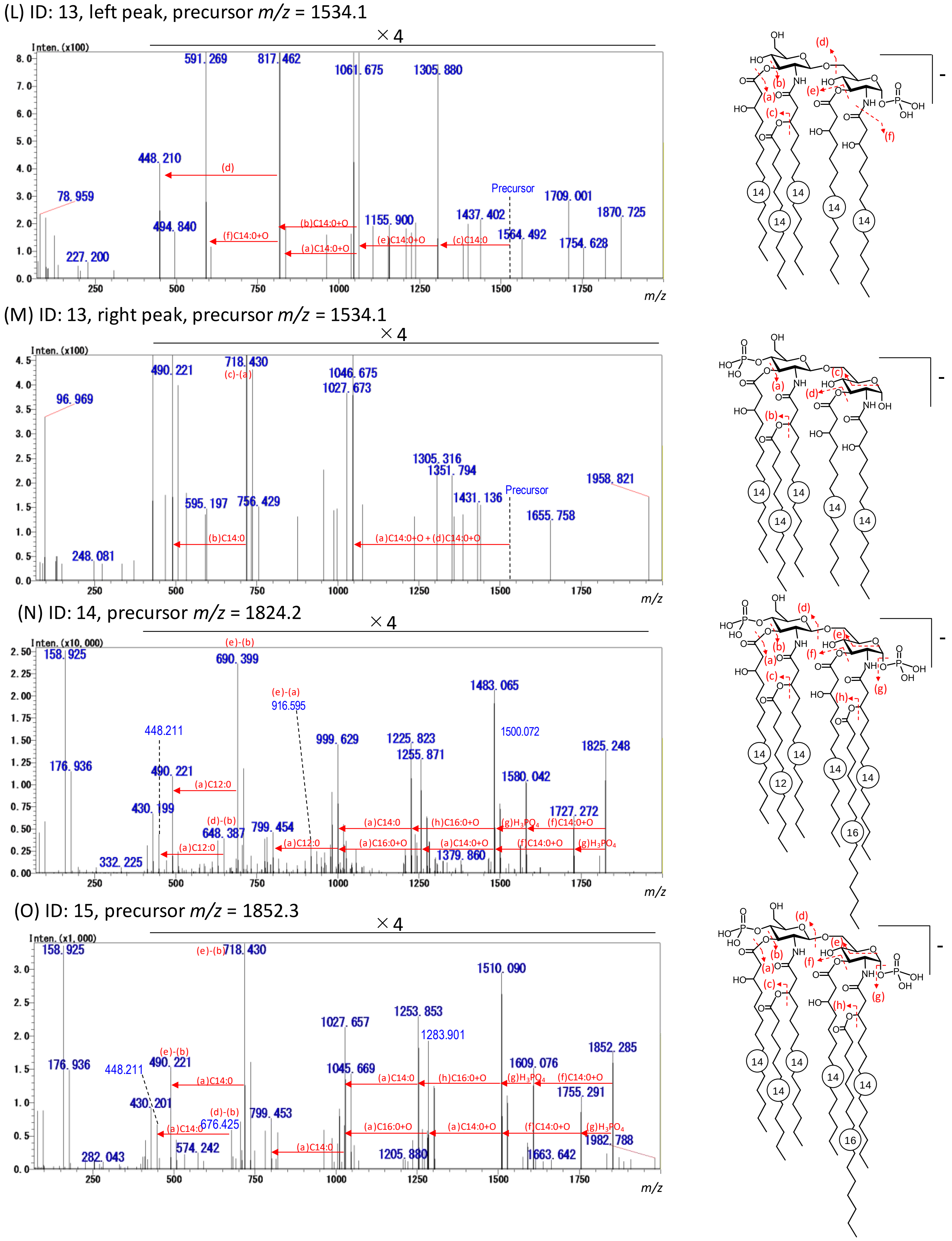

Figure S1. (Continued) MS/MS spectra of diverse molecular species present in *E. coli* lipid A.

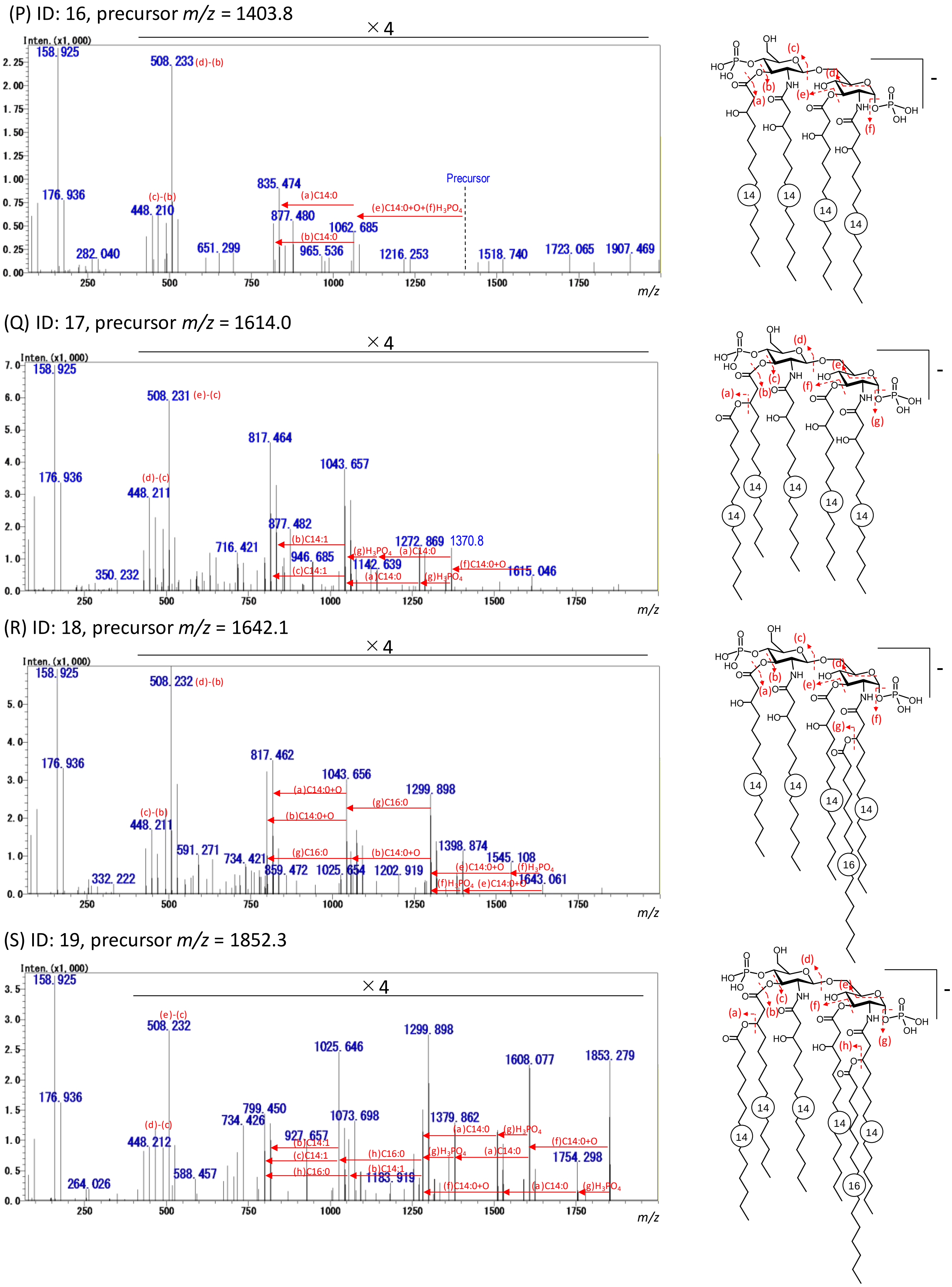

Figure S1. (Continued) MS/MS spectra of diverse molecular species present in *E. coli* lipid A.

(T) ID: 20, precursor m/z = 1850.3

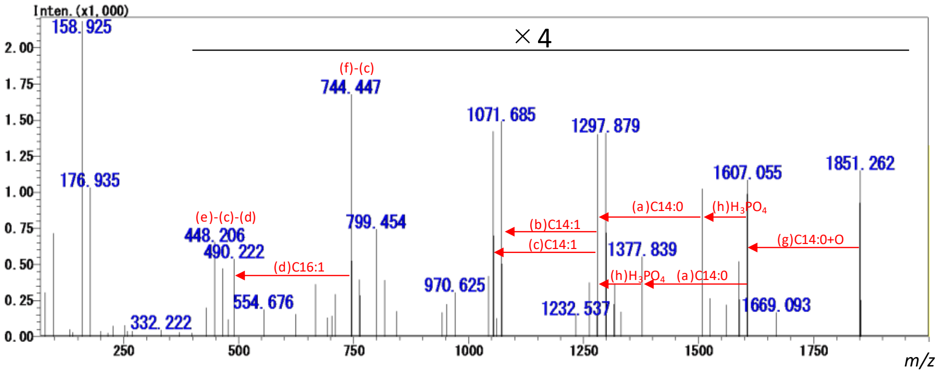

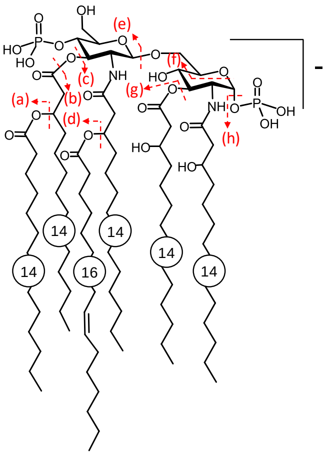

Figure S1. (Continued) MS/MS spectra of diverse molecular species present in *E. coli* lipid A.

(A) *B. thetaiotaomicron* lipid A 81:0

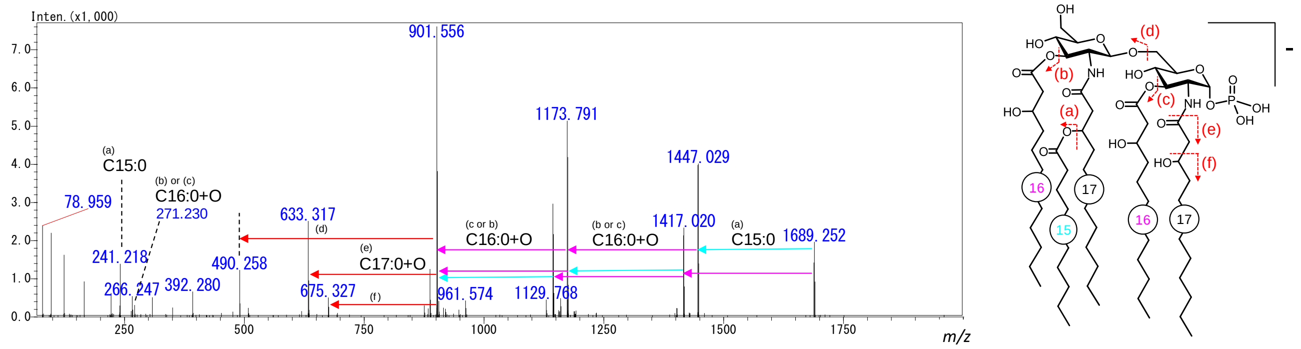

(B) *B. intestinalis* lipid A 81:0

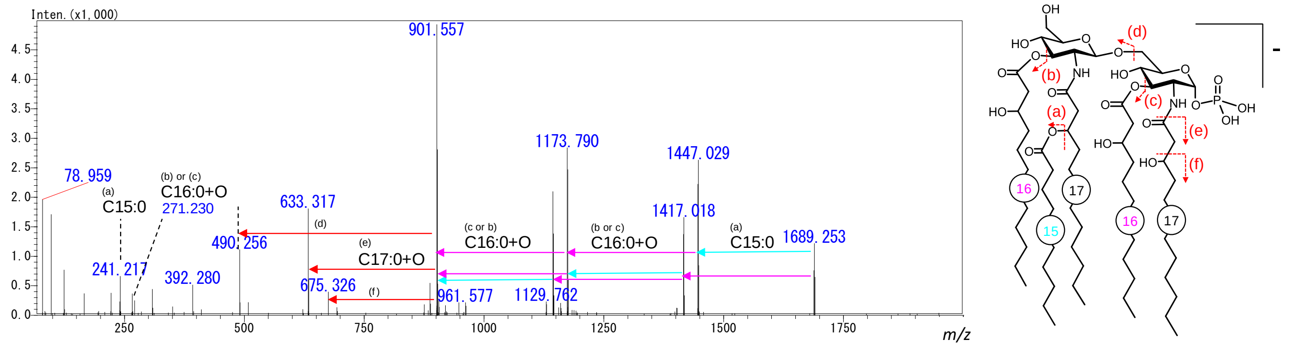

(C) *B. uniformis* lipid A 81:0

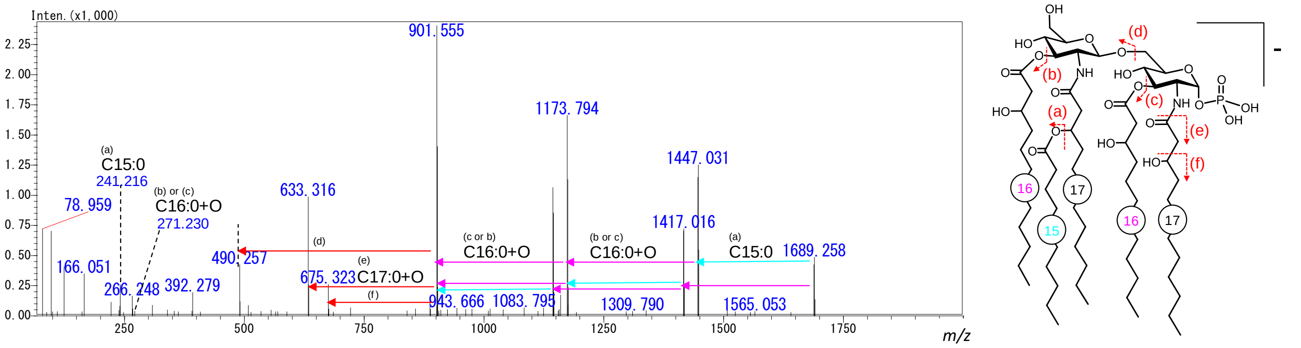

(D) *P. johnsonii* lipid A 81:0

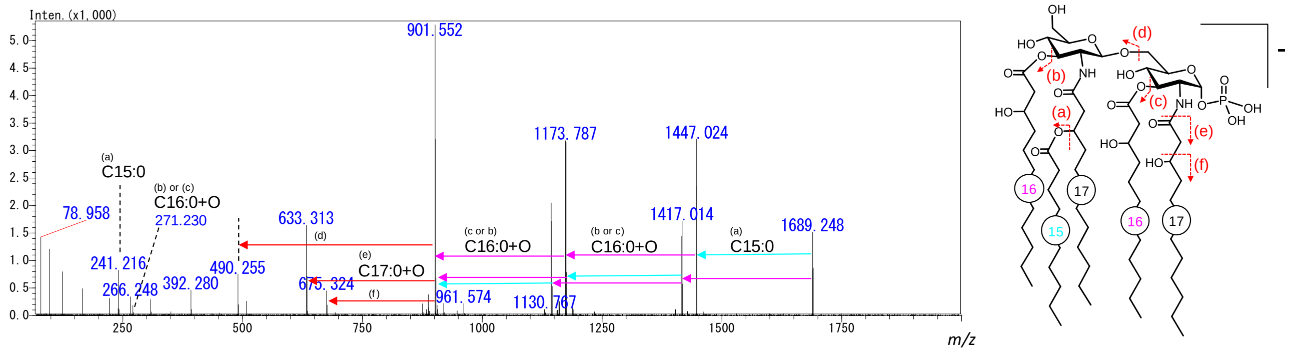

Figure S2. MS/MS spectra of lipid A 81:0 from Bacteroidetes strains.

(E) *P. xylaniphila* lipid A 81:0

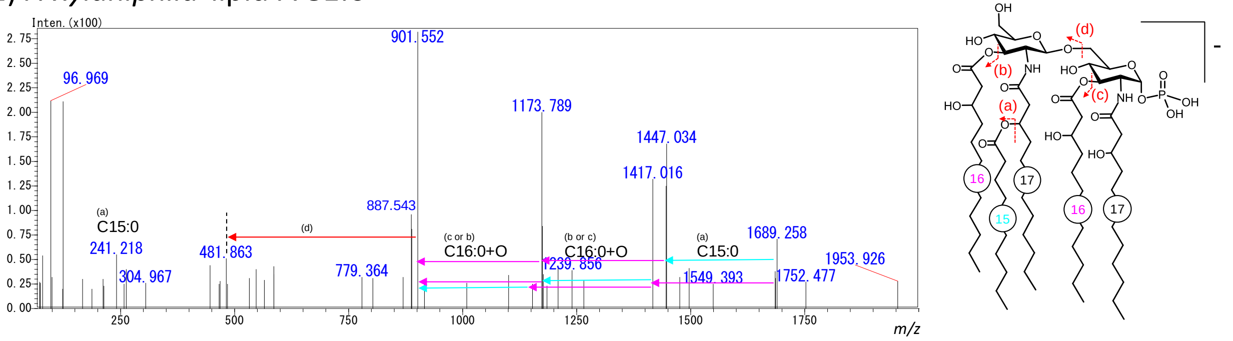

(F) *B. vulgatus* lipid A 81:0

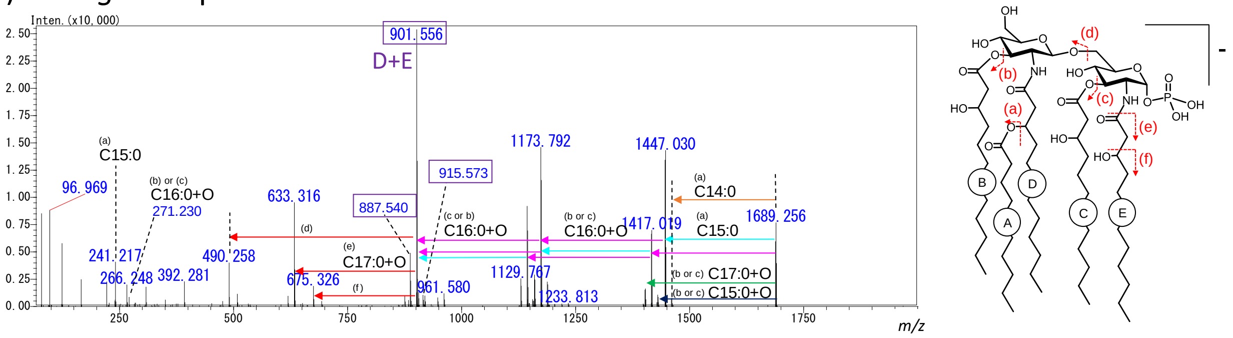

A = 14 or 15
D, E = 16 or 17 (D+E = 33, 34 or 35)
B, C = 15, 16 or 17 (B+C = 81–A–D–E)

(G) *O. laneus* lipid A 81:0

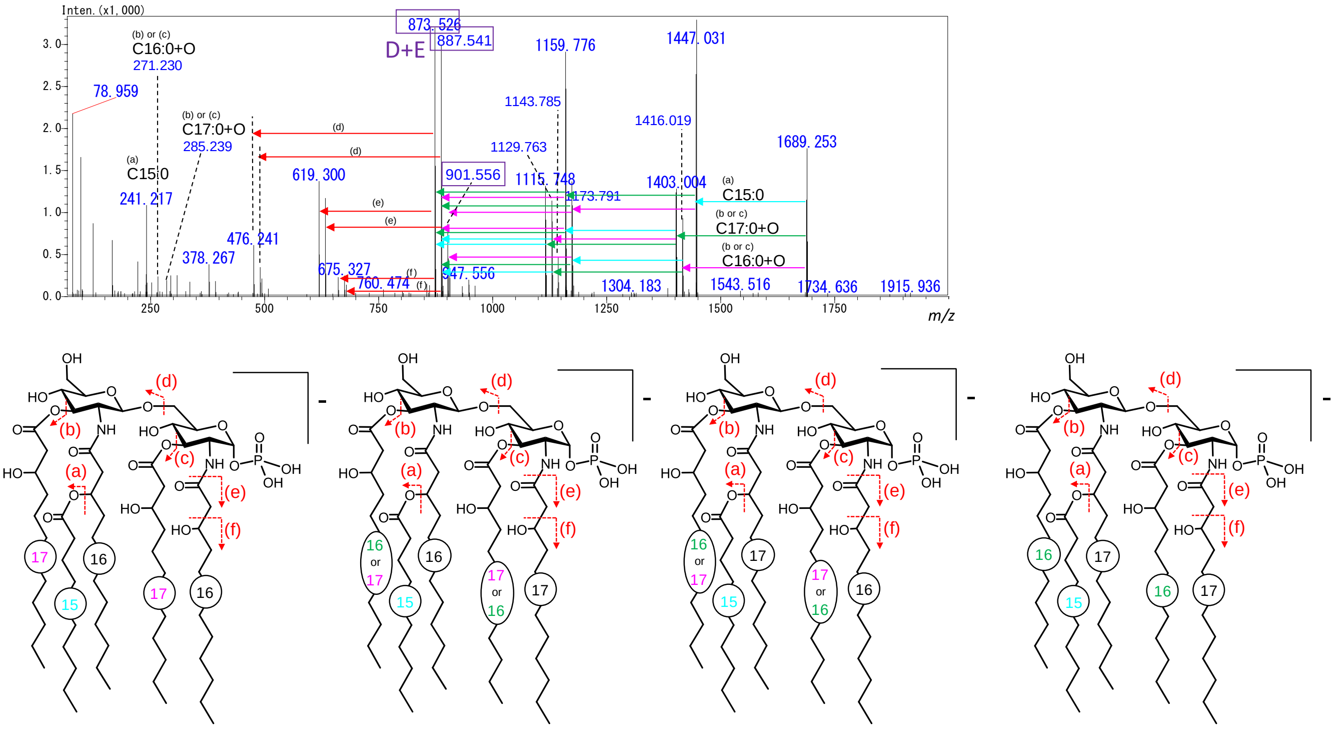

Figure S2. (Continued) MS/MS spectra of lipid A 81:0 from Bacteroidetes strains.

(H) *P. copri* lipid A 81:0

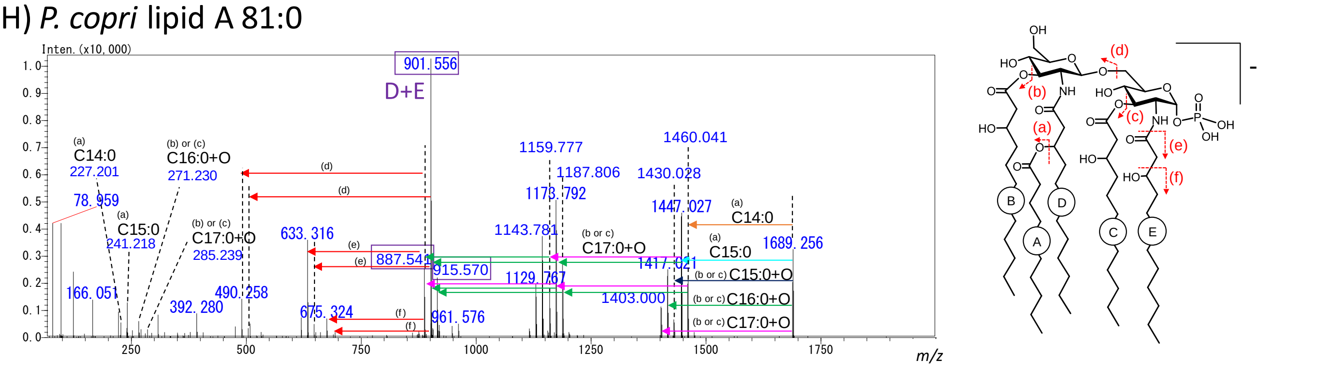

A = 14 or 15

D, E = 16 or 17 (D+E = 33, 34 or 35)

B, C = 15, 16 or 17 (B+C = 81–A–D–E)

Figure S2. (Continued) MS/MS spectra of lipid A 81:0 from Bacteroidetes strains.

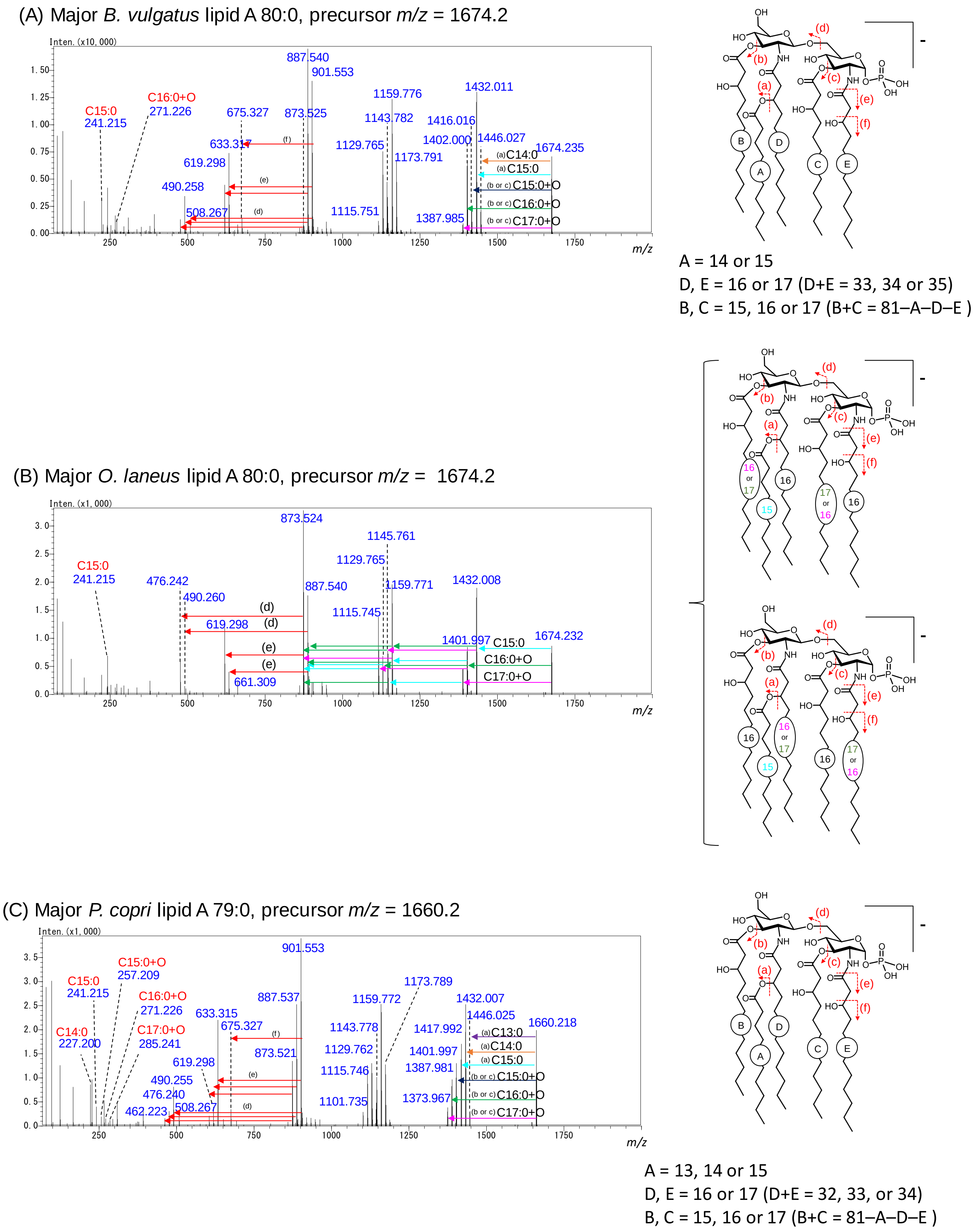

Figure S3. MS/MS spectra of dominant lipid A species in each Bacteroidetes strain.

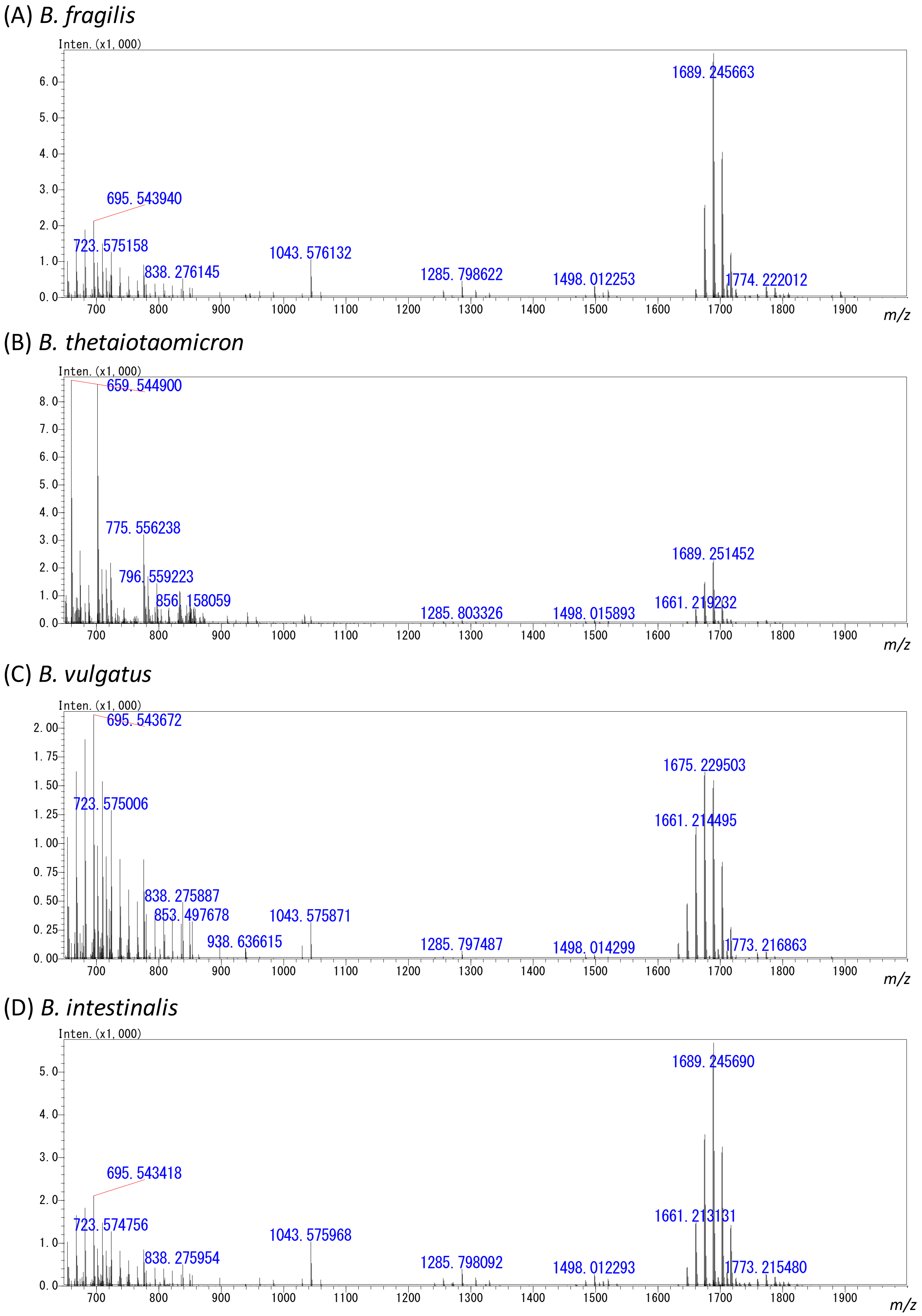

Figure S4. MS1 spectra of lipid A extracts in each Bacteroidetes strain. Average MS1 spectra of RT=10–12 min were presented.

(E) *B. uniformis*

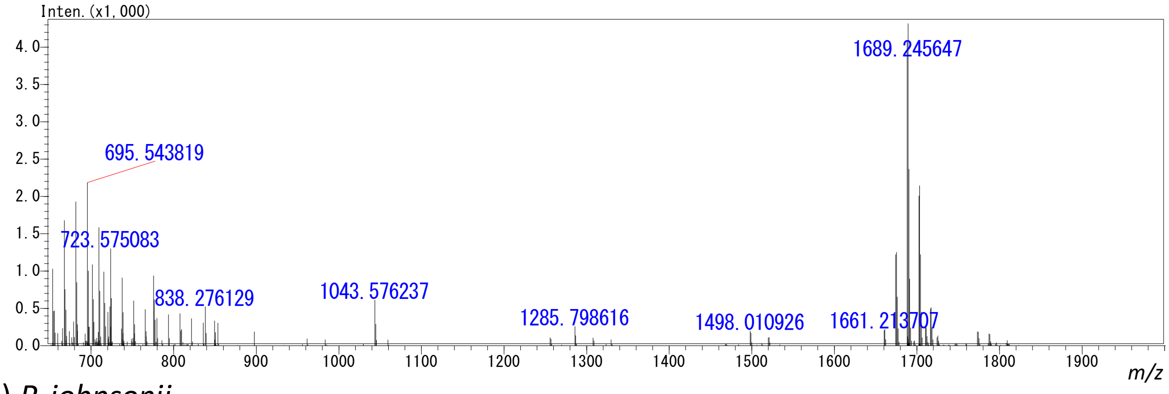

(F) *P. johnsonii*

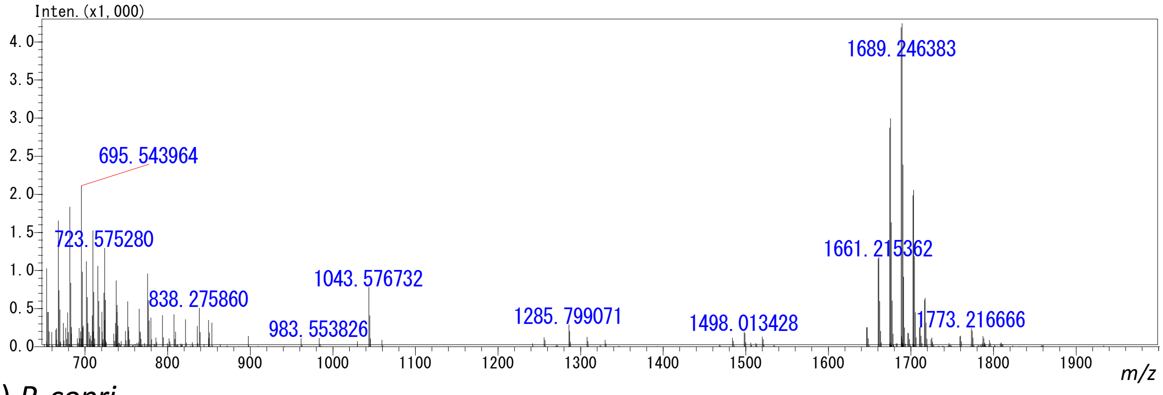

(G) *P. copri*

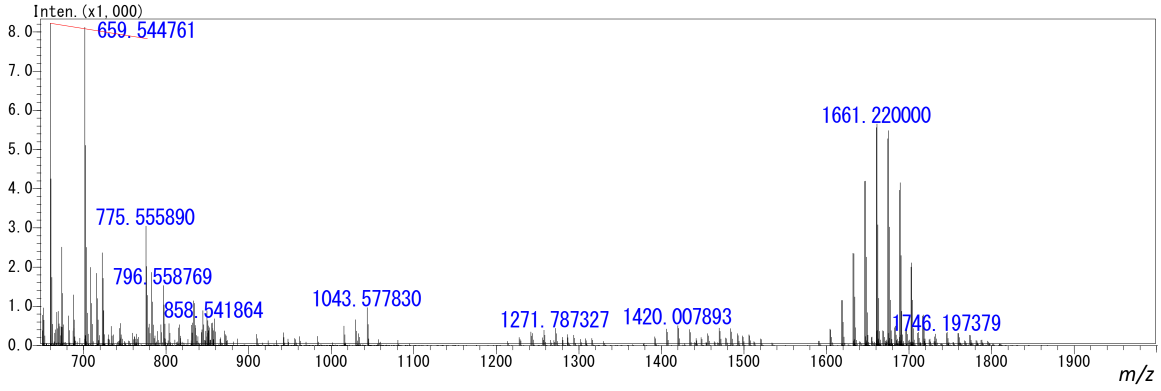

(H) *P. xylaniphila*

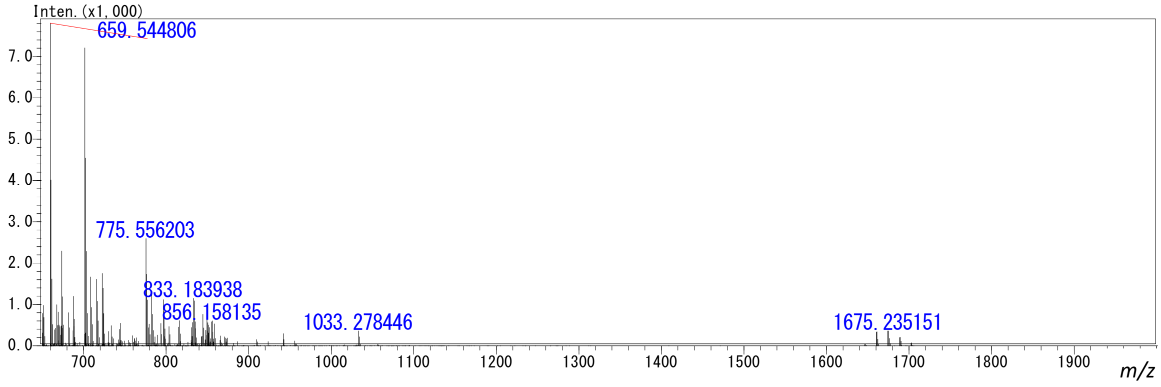

Figure S4. (Continued) MS1 spectra of lipid A extracts in each Bacteroidetes strain. Average MS1 spectra of RT=10–12 min were presented.

(I) *O. laneus*

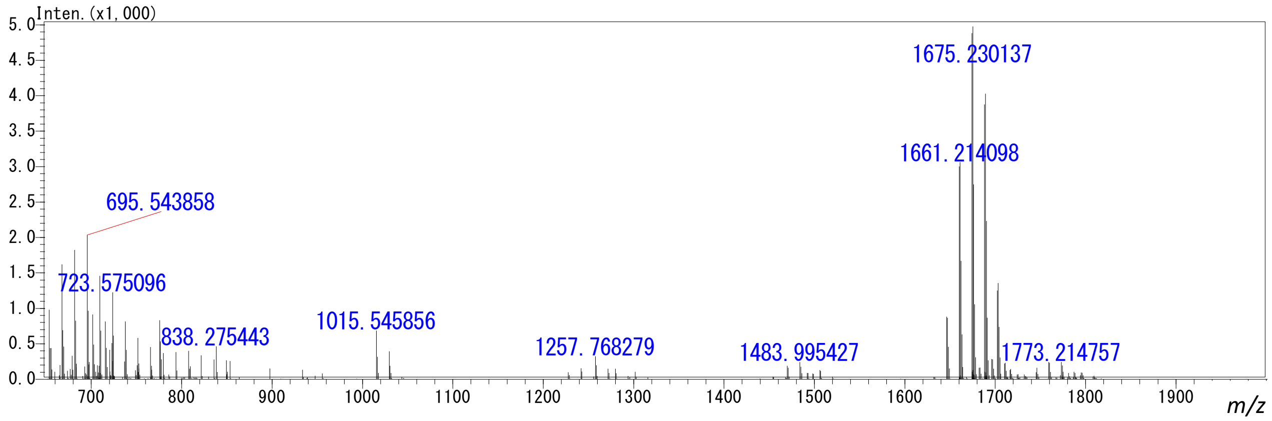

Figure S4. (Continued) MS1 spectra of lipid A extracts in each Bacteroidetes strain. Average MS1 spectra of RT=10–12 min were presented.
